# How the economic situation moderates the influence of available money on compulsive buying of students - A comparative study between Turkey and Greece

**DOI:** 10.1556/JBA.3.2014.018

**Published:** 2014-08-26

**Authors:** ALEXANDER UNGER, JULIE PAPASTAMATELOU, ELIF YOLBULAN OKAN, SINEM AYTAS

**Affiliations:** ^1^University of Applied Sciences, Ludwigshafen, Germany; ^2^Yeditepe University, Istanbul, Turkey

**Keywords:** compulsive buying, compensatory buying, economic crisis in Greece, economic boom in Turkey, available money of students, behavioral addiction, German Compulsive Buying Scale (GCBS)

## Abstract

*Background and aims:* Few studies about compulsive buying consider the economic framing situation. This study is concerned with the impact of different economic environments - the crisis in Greece vs. the boom in Turkey - on compulsive buying tendencies of students, while taking the role of gender and available money into account. *Methods:* Compulsive buying was measured by a Greek and Turkish translation of the German Compulsive Buying Scale (Raab, Neuner, Reisch & Scherhorn, 2005) in Greece and Turkey, which enabled an identification of compulsive and compensatory buyers. The questionnaires were administered to 119 Turkish and 123 Greek students (n = 242) enrolled in several universities in Athens and Istanbul. The data collection was conducted in a controlled and standardized way, namely in group-sessions lasting about 5 minutes, which were conducted and supervised by co-workers of the involved universities. *Results:* The results have shown that the percentage of compensatory buyers, but not compulsive buyers, within the Greek students sample was significantly smaller than within the Turkish student sample. Further as assumed the moderation of the economic situation could be confirmed: More available money only has a facilitating effect on compulsive buying tendencies under a positive economic environment. *Conclusions:* Anticipations about the financial situation and the general economic climate are more relevant for compulsive buying tendencies than one’s actual available money. Compensatory, but not compulsive buying was significantly smaller under crisis.

## Introduction

In a modern society, shopping symbolizes gratification, social status and autonomy. The symbolic function of shopping is communicated by mass media through advertising (cf. e.g. Elliott, 1997; Mikolajczak-Degrauwe & Brengman, 2014). One is not buying *Pepsi* only to quench one’s thirst, but also to express “*The choice of a new generation”.* One owns a *Mercedes* not only to drive from A to B, but also to communicate a certain socio-demographic status and success (Chao & Schor, 1998; Han, Nunes & Drèze, 2010); or for compensatory reasons, if one’s ego is threatened (Sivanathan & Pettit, 2010). Marketing strategies such as special offers, sales, discounts and loyalty cards also have an influence on consumers’ behavior. These strategies suggest that by buying these products, one becomes special and outstanding (Schindler, 1989).

Compulsive buying has been characterized in psychology as an irresistible urge to buy, with some form of tension relief or gratification (usually temporary)following the purchase (Workman & Paper, 2010). It is often accepted as an impulse control disorder (ICD), i.e., a dysfunctional consumer behavior with the following characteristics: 1)frequent preoccupation with shopping or irresistible buying impulses; 2)buying more than is needed and/or can be afforded; 3)distress related to buying behavior; 4)significant interference with work or social areas of functioning (McElroy, Keck, Pope, Smith & Strakowski, 1994). Other authors question a definite classification as an ICD and report a strong association of compulsive buying with obsessive compulsive disorder (OCD) symptoms (e.g. Frost, Steketee & Williams, 2002); but also, the possible assignment to the category of OCD remains problematic (cf. e.g. Black, Shaw & Blum, 2010). Ridgway, Kukar-Kinney and Monroe (2008) even emphasize that compulsive buying may feature components of both constructs. Another debated issue related to this concerns the assumed uncontrollability of compulsive buying, which has been questioned and criticized by Nataraajan and Goff (1991). These authors suggest that only a very small group of compulsive buyers can indeed not control their purchasing behavior.

This study examines the consumption behavior among Turkish and Greek students. Turkey demonstrates high consumption patterns, and can be characterized as a booming threshold country quite unimpaired by the world-wide financial and economic crisis of the last years. The OECD (2012, p. 200)prognosis for Turkey implies an annual average growth rate of 5.2% during 2012-2017, which means that Turkey will be one of the fastest growing OECD-members. The opposite holds true for Greece, which is currently experiencing a serious financial and economic crisis, accompanied by severe sociological and psychological consequences.

While both countries have a relatively high consumer culture, the difference in their actual economic development allows for the testing of the impact of the economic environment on the prevalence of compulsive buying. The fact that compulsive buying can be characterized by peaks, as well as by less active phases indicates that besides personal causes such as low self-esteem or negative emotions, external factors like the economic climate may play an essential role.

We hypothesize a decline of the overall compulsive buying tendency in Greece compared to Turkey, but with different reasons regarding compulsive and compensatory buying: If compulsive buying is indeed highly uncontrollable, then the percentage of Greek compulsive buyers should approximately be the same as in Turkey; whereas the percentage of compensatory buyers in Greece should be lower than in Turkey because these consumers can still control their consumption patterns and are able to react to Greece’s deteriorated economic situation.

Besides religion both countries show many similarities which concern the prevalence of a high consumption culture and a traditional social structure of tied family bonds. An analysis of the so called cultural dimensions by Hofstede (2013)shows many similarities. In three out of four available dimensions of this model, similar scores have been observed regarding “Power Distance” (60 in Greece vs. 66 in Turkey), “Individualism” (35 in Greece vs. 37 in Turkey)and “Masculinity/Femininity” (57 in Greece vs. 45 in Turkey). Merely according to “Uncertainty Avoidance”, the scores for Greece (112)clearly show a higher tendency compared to Turkey (85). Furthermore, both countries show a pronounced consumption culture, which is being regarded as responsible for pathological buying by interacting with certain personal factors such as depression (Lejoyeux, Tassain, Solomon & Adès, 1997; Mendelson & Mello, 1986; Nathan, 1988), low self-esteem (Faber & O’Guinn, 1992; Hirschman, 1992; O’Guinn & Faber, 1989; Scherhorn, Reisch & Raab, 1990) or impulsivity (Billieux, Rochat, Rebeteza & van der Linden, 2008; Black, 2007).

The above mentioned similarities of the two cultures enable the testing of the impact of the actual differing economic situations on tendencies of compulsive buying. In doing so, we seek to investigate two aspects: First, we assume that income, respectively available money, has an influence on compulsive buying. Second, we investigate in which way the economic framing situation is essential for the influence of available money on compulsive buying tendencies.

### Theoretical background

Typically, compulsive buying has been developing in a creeping manner. Compulsive buyers focus mainly on the process of buying itself and not on the bought items (Solomon, 2004). They start to buy to compensate for deficiencies (Scherhorn, Reisch & Raab, 1991, p. 24), which include frustrations due to unsolved private or professional problems, feelings of mental emptiness or meaningless and perceived non-acceptance by others (Lange, 2004, p. 132). Compensatory buying, which according to Lange (2004) could be understood as an unconscious strategy for self-regulation, could become compulsive buying in the long run, if the behavioral pattern becomes uncontrollable, which often could lead to overspending (Lo & Harvey, 2012).

### Causes and moderators of compulsive buying

#### The role of gender

Many previous studies have yielded interesting results on gender-based factors related to compulsive buyers. Dittmar, Long & Meek (2004)reported emotion- and identity-related dimensions of shopping to be more important for women than men. O’Guinn and Faber (1989) as well as Dittmar (2005) found that women tend to score higher than men as compulsive buyers. Thus, our first hypothesis concerns the hypothesized higher proneness of females to compulsive buying.

H1: Females will show a higher overall compulsive buying index.

H2: Females will show a higher percentage of compulsive buying.

#### Economic environment

The recent economic development of Turkey is characterized by a strong boom during the last 10 years, whereas Greece has suffered since 2008/09 from the worldwide economic and financial crisis. The per capita income increased constantlyto 10,605US Dollars in 2011, and 10,666 in 2012 (World Bank, 2013). In Greece there was a constant decrease of the per capita income between 30,399 US Dollars in 2008, to 22,083 US Dollars in 2012 (World Bank, 2013).

The hypotheses 3, 4 and 5 refer to differences between Turkey and Greece. We assume that the actual economic situation of a society has an essential influence on compulsive buying tendencies, although those who are indeed compulsive buyers (scoring 45 and above on the compulsive buying scale)should not be affected. A critical economic situation results in enhanced parsimony and reduced spending behavior. Thus, we assume reduced *compensatory buying* tendencies in Greece. Besides, we assume that an influence of money available (including own income as well as financial support by the family or state)will be moderated by the economic situation of each country, i.e., money available will have an influence in a boom situation, but not in a crisis situation. Or in other words, individual money available will have an influence in the absence of a crisis, but it will play no role if the economic situation is precarious. This could be attributed to the enhancement of fear and uncertainty avoidance in the case of a crisis. In this situation, the basic needs are foregrounded, while more post-materialistic needs are less important. In summary, we hypothesize that *compensatory buying* as well as the *overall compulsive buying tendency index* (which measured the tendency of all participants, including non-pathological and compensatory buyers) will be lower in Greece.

H3: The overall compulsive buying index will be higher in Turkey compared to Greece.

We further suggest that even in the face of decreased per capita income, the percentage of compulsive buyers in Greece will not have decreased. This suggestion is based on the assumption that *compensatory* buyers could reduce their purchasing behavior, whereas *compulsive* buyers could not reduce their purchasing behavior under critical macro-economic conditions.

H4: The percentage of compensatory buyers will be lower in Greece compared to Turkey.

H5: The percentage of compulsive buyers in Greece will be the same compared to Turkey.

The influence of income and available money on proneness of compulsive buying is important for the way in which the differing economic framing situations affect compulsive buying. Studies, which are concerned with this question, show inconsistent findings. O’Guinn & Faber (1989) as well as Scherhorn et al. (1990)report no influence of income on compulsive buying; whereas d’Astous & Tremblay (1989)argue that the relationship between the amount of income and compulsive buying has to be characterized as a u-turned function. However, according to the study by d’Astous, Maltais & Roberge (1990)on young consumers, there is no relation between their compulsive buying tendencies and the social status of their parents. In contrast to this, a moderate positive correlational relationship between the amount of income and compulsive buying is reported for Turkey (Ergin, 2010). In sum, the results towards the influence of income or available money are contradictory.

The not yet clarified role of income raises the suggestion that there may be a fluctuation in the function and importance of money. One aspect of this consideration - the role of the economic framing situation - is analyzed in the current study. We assume that the influence of available money (income and others)on compulsive buying depends on the actual economic framing situation. It is assumed that the perceived function of money changes if the economic framing situation differs, as in the case of Turkey and Greece. Several authors report enhanced materialistic values and the importance of modern consumption culture for compulsive buyers (Belk, 1985, 1999; Dittmar, 2005; Neuner, Raab & Reisch, 2005; Raab, 2000). The new possibilities of internet purchases may also reinforce compulsive buying tendencies (Rose & Dhandayudham, 2014).

The present study suggests that the assumption about materialistic values and the symbolic meaning of money is only true within stable, mature industrial societies or in booming threshold countries (like Turkey). If consumers have to cope with an essential financial crisis, then the perceived meaning of money is fundamentally changed; since according to the hierarchy of needs by Maslow (1943), consumers have to satisfy lower level basic needs before progressing on to meet higher level growth needs. In addition, they have to cope with an increased feeling of uncertainty and ambiguity. Inglehart & Abramson (1994)developed a model on the basis of the hierarchy of needs, examining the relationship between economic security and value change. “[They] contend that only when the satisfaction of the materialistic survival needs can be taken for granted (e.g., due to financial or economic stability and job security), the focus on need fulfilment will gradually shift to higher ‘Postmaterialist’ goals […]” (Miesen, 2012, p. 221).

In accordance with these considerations, it is assumed that the prevalence of the materialistic meaning of money and its function as a symbol for success and well-being in life will be alleviated in the Greek sample. Thus, the economic framing situation will moderate the influence of available money on compulsive buying tendencies; and in Greece, the amount of available money will have no influence because its functional meaning has been altered by the crisis and will be downplayed to the now important function of simple, but fundamental economic needs. In Turkey, an increase in available money, however, will lead to increased compulsive buying tendencies; because of the current economic booming phase, the influence of materialistic values could enhance the consumption and reinforce compulsive buying tendencies. Under these conditions, higher income and available money enable more possibilities to engage in pathological buying, especially for those who seek to compensate perceived personal shortcomings such as low self-esteem, feelings of and those affected by depression, feelings of guilt and worthlessness, emptiness and feelings of indifference (cf. Faber & Vohs, 2004). Based on the above discussed considerations, we formulate the following hypothesis:

H6: The influence of money available on compulsive buying tendencies will be moderated by the economic situation: the influence will be higher in economic boom (Turkey)compared to a financial crisis situation (Greece).

## Method

### Measures

We have measured the compulsive buying tendency through both a Turkish and Greek translation of the German Compulsive Buying Scale (GCBS, cf. Raab et al., 2005). For both versions, the back-translation method was used. Inconsistencies were carefully modified and adapted. This 16-item and 4-point measurement tool ranging from “I don’t agree” (1)to “I totally agree” (4), allows a classification of consumers as inconspicuous, compensatory or compulsive buyers based on cut-offs on the scale. The cut-off score for being at risk for compensatory buying is 35.5 points and for compulsive buying is 44.6 points on the scale (cf. Raab et al., 2005). All of the items were summed up to an overall compulsive buying index ranging from 16 to 56, with the higher values indicating a higher proneness. Individuals scoring higher than 44 were identified as being at high risk for compulsive buying and were classified as “compulsive buyers”. Those individuals scoring between 36 and 44 were identified as “compensatory buyers”, they showed enhanced buying behavior, but they could still control their buying behavior; thus it could be understood as an intermediate level between inconspicuous and compulsive buyers (Friese, 2009). The measurement tool captures different aspects of compulsive buying like impaired impulse control (e.g. item 3: *I often have an unexplainable urge, a sudden and spontaneous desire, to go and buy something*), post-purchase regret (e.g. item 6: *After making a purchase I often ask myself if this purchase was really necessary*)and irrational money spending (e.g. item 7: *I often buy something, simply because it is cheap).* Cronbach’s alpha was .80 for the Turkish, and .85 for the Greek subsample. This validated measurement tool has been used in several German studies (Neuner et al., 2005; Raab, Reisch & Unger, 2010; Scherhorn, Reisch & Raab, 1992), in other European countries like Denmark (Reisch, Gwozdz & Raab, 2011)and is based on a former Canadian version (Valence, d’Astous & Fortier, 1988). Further, we measured the monthly available money of the students. The exact wording of this item was: *How much money is in total available for you per month (Including your own income, family support, governmental financial support like grants or others)?*

The sample consisted of Turkish (*n* = 119) and Greek (*n* = 124) students in universities in Istanbul and Athens, respectively. The gender ratio was the same in both samples (56.8% in Turkey and 56.9% in Greece were females). The mean age of the Turkish sample was *M* = 22.93 years; *SD* = 3.18 and the one for the Greek sample was *M* = 23.50 years; *SD* = 3.41. This difference reached no significance, *t* (221) = -1.30; *p* = .196). In order to test the influence of the economic situation as a moderator variable on the influence of available money on compulsive buying tendencies, it was necessary for both samples to show no significant differences in available money. For such a comparison, we could not use the z-standardized values of available money on both corresponding subsamples, as in the case of the moderator analysis. So we calculated the value of the Turkish Lira into the Euro based on the currency course of March 22, 2013, which was the temporal end point of our data collection in Turkey and Greece. No significant mean difference in available money was observed, *t* (228) = 1.15 ,*p* = .253 (M_TURKEY_, 487.86 EUR; *SD* = 316.68 vs. M_GREECE_= 436.82 EUR; *SD* = 454.24). One may wonder how it is possible that both samples showed no differences in monthly available money, even though one sample suffers from crisis and the other profits from the current boom. Our post-hoc explanation of a counter effect in both countries is that Greek students have received much more support from their families during the crisis (many Greek students live with their parents). On the other side, the Turkish students have not (yet) profited from the macro-economic boom situation to the same extent as regular employees or business persons would have. For our purpose, this enabled us to test the effect of the macro-economic situation.

### Procedure

The data collection was executed by group-sessions of between 5 and 10 students in different universities in Istanbul and Athens. It was ensured that each participant filled out the questionnaire undisturbed. All sessions lasted about 5 minutes and were conducted by local coworkers of the cooperating institutions.

### Statistical analysis

To test the hypotheses, we conducted an analysis of variances (ANOVA), binary logistical regressions and linear regressions including a moderator analysis. We used binary logistical regressions, which is a method for testing the influence of one or several dichotomous or multi-stage level independent variables on a dichotomous coded dependent measurement; that is, in our study to be scored as either a compensatory or compulsive buyer, respectively. A binary logistical regression allows for testing models with a dichotomous dependent variable. The revealed effects could be interpreted as probabilities and recalculated into percentages.

### Ethics

Ethical standards were granted by the approval of the institutional review boards of Yeditepe University, Istanbul, Turkey and Panteion University, Athens, Greece. All subjects were provided information about the study. Further, informed content was signed by all participants.

## Results

Hypotheses (1)and (3)were tested by an ANOVA on the overall compulsive buying index. As assumed, we observed a main effect of gender, F(1, 224)= 22.15, *p* < .001; partial ŋ^2^ = .09, as well as of country, f(1, 224)= 25.15, *p* < .001; partial ŋ^2^ = .10. The compulsive buying index was higher in Turkey (M_TURKEY_ = 36.94; *SD* = 7.27) compared to Greece (M_GREECE_ = 31.78; *SD* = 8.51). Furthermore, women (M_WOMEN_ = 36.13; *SD* = 8.98) showed a higher compulsive buying tendency than men (M_MEN_ = 31.65; *SD* = 6.65). The interaction of gender by country did not reach significance; F(1, 224)= 0.30, *p* = .586; partial ŋ^2^ = .001 (Table 1).

Hypotheses (2), (4)and (5)were tested by a binary logistical regression which included gender and country as factors, and available money as a standardized covariate.

As predicted, only gender had an influence on the probability of being a compulsive buyer, *B* =1.93, *p* = .004 (Table 2). Women were more affected in the overall sample. The factor country showed no difference, *B* = -0.79, *p* = .238. The same was observed for the interaction of country by gender, *B* = 0.22, *p* = .868. The covariate, available money reached a significant influence in a positive direction in the overall sample, *B* = 0.40, *p* = .045 (Table 2).

**Table 1.** Two-way analysis of variance (country by gender and available money as a z-scored covariate on the Compulsive Buying Index

Source	*df*	*SS*	*MS*	*F*	*p*	*η*2
Available money	1	455.47	455.47	7.99	.005	.034
Country	1	1,434.48	1,434.48	25.15	<.001	.101
Gender	1	1,263.36	1,263.36	22.15	<.001	.090
Country by gender	1	16.95	16.95	0.30	.586	.001
Within cells	224	12,774.06	57.03			
Total	229	283,966.02				

**Table 2.** Binary logistical regression “country and gender” predicting percentage of compulsive buying

Variable	*B*	*SE*	*OR*	95% CI	Wald statistic	*P*
Country (1) by gender (1)	0.22	1.34	1.25	[0.09, 17.31]	0.03	.868
Country (1)	-0.79	0.67	0.45	[0.12, 1.69]	1.39	.238
Gender (1)	1.93	0.67	6.90	[1.84, 25.80]	8.23	.004
Available money (cov)	0.40	0.20	1.49	[1.01, 2.20]	4.02	.045

*Note:* For country Turkey was chosen as reference category (Turkey = 1; Greece = 2); for gender male was chosen as reference category (males = 1; females = 2); available money was entered as a continuous variable.

**Table 3.** Binary logistical regression “country and gender” predicting percentage of compensatory buying

Variable	*B*	*SE*	*OR*	95% CI	Wald statistic	*p*
Country (1) by gender (1)	-0.40	0.65	0.67	[0.19, 2.39]	0.38	.539
Country (1)	-0.69	0.32	0.50	[0.27, 0.94]	4.58	.032
Gender (1)	1.34	0.33	3.82	[2.01, 7.28]	16.62	<.001
Available Money (cov)	0.30	0.16	1.34	[0.98, 1.84]	3.38	.066

*Note:* For country Turkey was chosen as reference category (Turkey = 1; Greece = 2); for gender male was chosen as reference category (males = 1; females = 2); available money was entered as a continuous variable.

**Table 4.** Regression analysis for country (z-standardized) and available money (z-standardized) predicting compulsive buying tendency (overall sum index) without and with P (Moderator)

Variable	*B*	*SE B*	*β*	*t*	*p*
*Model without moderator*					
Z-score (country)	-2.61	0.52	-.31	-5.01	<.001
Z-score (available money)	1.21	0.52	.14	2.31	.022
*Model with moderator*					
Z-score (country)	-2.61	0.52	-.31	-5.06	<.001
Z-score (available money)	1.26	0.52	.15	2.41	.017
P (country by available money)	-1.21	0.52	-.15	-2.34	.020

*Note:* The moderator P is calculated by the multiplication of Z-score (country) and Z-score (available money).

The same significant proneness for women was observed, when the dependent measurement compensatory buyer was analyzed, *B* =1.34, *p* < .001. In contrast to the binary regression for compulsive buyers, the factor country showed a significant effect on the compensatory buying probability in the predicted direction, *B* = -0.69*,p* = .032. In Greece, the probability of being a compensatory buyer was significantly reduced compared to Turkey. The interaction of country by gender reached no significance, *B* = -0.40, *p* = .538 and the covariate reached significance at the 10% level, *B* = 0.30*,p* = .066 (Table 3). The percentages were for compensatory buyers 37.0% (Turkey) compared to 26.2% (Greece), whereas the corresponding probabilities for the non-significant difference for compulsive buying were 13.4% (Turkey) versus 7.3% (Greece). To test hypothesis (6), we conducted a corresponding moderator analysis (Table 4).

**Figure 1.**
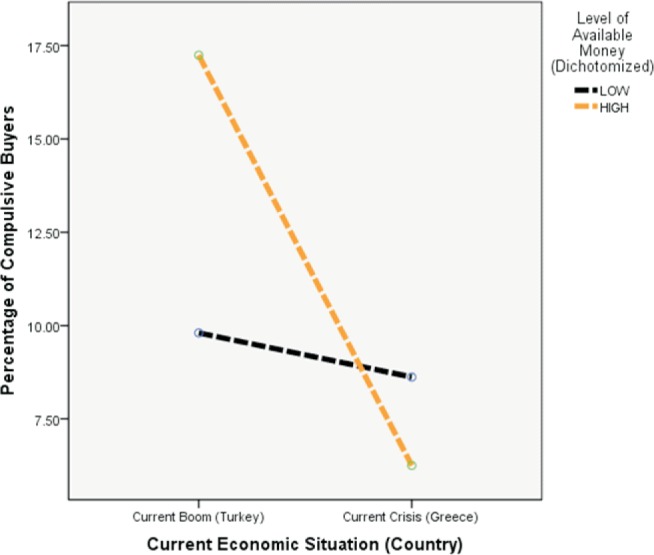
Percentage of compulsive buyers by available money and current economic situation

The moderator factor reached significance, ß = -0.15, *t*(226) = -2.34*,p* = .020. For the coding, Greece = 2 and Turkey = 1, respectively; the negative sign of P indicated that the moderating effect of country (current economic situation)implies that the influence of available money on compulsive buying decreased if Greece (“crisis”)instead of Turkey (“boom”)was regarded. The higher income group shows a significantly higher percentage of compulsive buyers in Turkey, but not in Greece (Figure 1).

For a specification we conducted two separate linear regressions on compulsive buying for Greece and Turkey. We repeated this by two further multiple regressions, including gender as an additional factor.

**Table 5.** Separate Linear Regression for Greece and Turkey by available money (Model 1) and by available money, gender and age (Model 2) on compulsive buying overall index

Variable	*B*	*SE B*	*β*	*t*	*p*
			Greece		
*Model 1* Available money	0.06	0.78	.01	0.08	.940
*Model 2* Available money	0.35	0.77	.04	0.46	.649
Gender	3.90	1.56	.23	2.51	.014
			Turkey		
*Model 1* Available money	2.48	0.66	.34	3.77	<.001
*Model 2* Available money	2.59	0.61	.36	4.24	<.001
Gender	5.41	1.23	.37	4.39	<.001

The results of both variations of the country-specific subsamples correspond to the prior overall-analysis (Table 5): In the case of the Turkish sample, we observed again a positive significant effect of available money, regardless of whether gender was introduced, ß = 0.36, *t*(105)= 4.24, *p* < .001 (Model 2)or not, ß = 0.34, *t*(107)= 3.77, *p* < .001 (Model 1). However, this effect could not be observed in the Greek subsample, ß = 0.01, *t*(119)= 0.08, *p* = .940 (Model 1). In the case of the introduction of gender, the effect of available money remains non-significant in the Greek sample, ß = 0.04, *t*(118)= 0.46, *p* = .649 (Model 2). Thus, the factor of available money showed, in accordance with the previous moderator analysis, an influence in Turkey, but not in Greece. The factor of gender reached significance in both subsamples, ß = 0.23, *t*(118)= 2.51, *p* = .014 for Greece and ß = 0.37, *t*(105)= 4.39, *p* < .001 for Turkey.

## Discussion

The purpose of the current study was to show the influence of the financial crisis versus the boom on compulsive buying tendencies in Greece and Turkey. In this context, we also tested the influence of available money and gender. The main results of our study can be summarized as follows: First, the hypotheses according to the higher compulsive buying index and higher percentage of compulsive buying for women were both confirmed. The general hypothesis of the high overall compulsive buying index in Turkey compared to Greece was supported by our data. This was qualified by higher percentages of *compensatory* buyers in Turkey and not significant differing percentages according to *compulsive* buyers. Finally, the moderator-analysis revealed that the influence of available money on the compulsive buying tendencies of the students was moderated by the economic macro-situation.

Our data underlines that not all Turkish students are in a financially safe situation, and not all Greek students are in a financially precarious situation. What we can take for granted, however, is that the economic situation differs in the outlined way between the two countries. This does, however, not imply that the boom in Turkey is unequivocally observable in all parts of the country, and it also does not imply that it is guaranteed that the boom in Turkey will continue. What is decisive in our argumentation is that the economic climate in both countries is clearly diverged currently in the assumed way. Several economic indicators justify our reasoning: A comparison of Gross Domestic Product (GDP)data shows that Turkey displayed higher economic growth between 2011 and 2013 (2011: 8.8, 2012: 2.2, 2013: 3.6 in Turkey vs. 2011: -7.1, 2012: -6.4, 2013: -3.5 in Greece). For the same years, lower unemployment rates were reported for Turkey (Turkey 2011: 9.6, 2012: 9.0, 2013: 9.4 vs. Greece 2011: 17.7, 2012: 24.2, 2013: 27.2)(cf. OECD, 2013a; 2013b). With respect to trade deficits (Trading Economics, 2014a, 2014b), a higher trade deficit - measured by the debt-to-GDP ratio - for Greece compared to Turkey was reported between 2011 and 2013 (Greece 2011: 148.3, 2012: 170.3, 2013: 156.9 vs. Turkey 2011: 42.2,2012: 39.4, 2013: 36). The abovementioned data indicates that while Turkey was also affected by the international financial and economic crisis, the situation in Greece was far more precarious.

Several methodological limitations of our study have to be considered. The use of convenient student samples could cause low external validity. Thus, our results are only restrictively transferable to the overall population. A testing of our hypotheses by using a representative sample of both countries would be the most straightforward test to enhance external validity. The self-report measure as well as the cross-sectional design of the study presumes comparability of the data and a testing of possible measurement invariance.

The adequateness of the sample size of 242 has to be considered. For testing for an adequate sample size, a g-power analysis could be a useful tool (Faul, Erdfelder, Buchner & Lang, 2009; Faul, Erdfelder, Lang & Buchner, 2007)1.^1^ One has to further consider the difference between *a-* and ß-error (e.g. Fiedler, Kutzner & Krueger, 2012). *a* -error implies that the alternative hypothesis is assumed as true but indeed it is not. The ß-error - the non-acceptance of an indeed valid difference - could be reduced by using a higher sample size at the expense of the a-error. In the given study, we report mostly significant differences, thus we could not commit a ß-error in these cases, whereas the possible a-error could not be reduced by higher sample sizes. Taking this into account, we can summarize that the used sample size is not necessarily too small according to testing the relationship between the economic situation (in Greece and Turkey)and proneness to compulsive buying; but it would, however, be too small if we sought to conduct representative studies for the whole populations of the two involved countries (as conducted for the case of Germany by Neuner et al., 2005). There could be another argument for higher sample sizes which arise from some other limitations of our study, which will be described next: One limitation may arise from possible cultural differences between both countries. Although, as outlined in the section ‘Economic environment’ both cultures show - beside religion - many similarities, therefore we highly recommend that future research control more for cultural differences to avoid the potentially imprecise assumption of a similar culture.

Further, it seemed straightforward to focus more on the specific detailed financial situation of the families and also on the individual optimism according to future professional prospects (as hopes and fears according to an anticipated professional career). Although we already asked for the available money of the participants, the financial situation should have been scrutinized in more detail (as differentiating between one’s own income, support by family members, including financial as well as indirect support by providing accommodation and food through parents or other close family members). Adding these factors may enable the testing of our results more rigorously by controlling for possible alternative explanations and disturbance variables. Of course the increased number of variables will then require enlarged corresponding sample sizes. Against this background, we recommend two types of studies for future research: First, replication studies with student samples that thoroughly consider the mentioned variables; and second, representative population studies - as conducted by Neuner et al. (2005) for Germany - to enlarge the scope to other parts of the populations besides students. For both types of studies, we recommend the cautious testing of higher sample sizes, but to use g-power analysis to choose an adequate sample size.

The measurement invariance problem may be another weakness of our study and it is strictly necessary to consider this problem in future research. This problem is also issued (cf. Horváth, Adigüzel & van Herk, 2013)for other scales such as the Compulsive Buying Scale (Faber & O’Guinn, 1992)and the Compulsive Buying Index (Ridgway, Kukar-Kinney & Monroe, 2008). Although our results as outlined have to be evaluated with a good dose of cautiousness, they nevertheless seem to issue the up to date, not very intensively considered aspect of the macro-economic situation. We think that it is worthwhile to further scrutinize the role of the economic macro-situation for compulsive buying in future research studies. These studies should, however, include as outlined above, a more elaborated and detailed measurement of the financial situation as well as those of perceived optimism or pessimism towards the anticipated financial situation, and thus consider possible confounding variables. This may enable the more precise detangling of the possible effects of one’s own financial situation, the macro-economic situation and those of the individual perceptions of both.

## Conclusions

This study has shown that the economic framing situation moderates the influence of available money in the predicted way. Available money leads to increased compulsive buying in Turkish students, but not in Greek students; although the individual financial situation of both samples was similar. So we could exclude alternative explanations, for example that different levels of available money in both samples could be responsible for the observed results. It seems plausible that as argued, the functional meaning of money is different during a period of financial crisis and under conditions of a booming economy, and thus, can alter the corresponding influence on compulsive buying tendencies. The results have shown that the percentage of compulsive buyers in Greece and Turkey is the same, whereas there is a significant decrease in Greece compared to Turkey regarding the overall compulsive buying tendency, including non-patho-logical and compensatory buyers. This finding seems to be more compatible with the behavioral addiction assumption: consumers suffering from compulsive buying presumably do not adjust their spending behavior according to changing economic circumstances, as the case of Greek compulsive buyers indicates. In contrast, the Greek inconspicuous and compensatory buyers are able to reduce their enhanced shopping behavior, and thus, showed significant decreased compulsive buying tendencies compared to their Turkish counterparts. This reasoning has, however, one important limitation: We assume as a perquisite that the Greek data before the crisis was comparably high compared to that of Turkey, according to the percentage of compulsive buyers. Indeed, we have had some indirect hints for the correctness of this assumption, like pronounced consumption culture and the policy of cheap private credit before 2008/09. The essential limitation is, however, that no figures about compulsive buying were available about compulsive buyers in Greece *before* the crisis. The high cultural similarities make the correctness of our reasoning probable, but due to the missing data we could not test this in an objective way. This again emphasizes the importance of future research, to study how the compulsive buying tendencies will be changed when the economic development in Greece is ameliorated.
